# The First Histological Analysis of the Tissues Lining the Fossa Navicularis: Insights to its Etiology

**DOI:** 10.7759/cureus.1299

**Published:** 2017-05-31

**Authors:** Sarvenaz Sheikh, Joe Iwanaga, Steven Rostad, Tarush Rustagi, Rod J Oskouian, R. Shane Tubbs

**Affiliations:** 1 Seattle Science Foundation; 2 Pathology, CellNetix; 3 Swedish Neuroscience Institute, Swedish Medical Center; 4 Neurosurgery, Complex Spine, Swedish Neuroscience Institute; 5 Neurosurgery, Seattle Science Foundation

**Keywords:** occipital bone, clivus, anatomy, histology, computed tomography, notochord, fossa navicularis

## Abstract

The fossa navicularis (FN) is an anatomical variant on the ventral surface of the basilar part of the occipital bone that, to date, has only been investigated in bone specimens. We aim to clarify the structure of the fossa navicularis by gross anatomical, radiological, and histological methods. The FN was found in the occipital bone of the Caucasian male cadaver. There was no bony or histological continuity between the FN and posterior cranial fossa. The histological analysis found that the overlying tissue was composed of loose connective tissue with a mixture of collagen and elastic fibers and a vascular matrix including arteries, veins, and capillaries. There was no evidence of lymphoid, glandular, or notochordal tissues. As no previous studies have performed histological analysis of the FN, this report adds to our knowledge of tissues that are involved in its formation.

## Introduction

The fossa navicularis (FN) is a small round depression [1] located on the ventral surface of the basilar part of the occipital bone and superior to the pharyngeal tubercle. Studies of this structure using a dry skull and computed tomography (CT) have been reported [2]. The etiology of these depressions has only been speculated until date. For example, some authors have posited that this fossa could be filled with nasopharyngeal tonsillar tissue [2-4]. However, to our knowledge, none of the previous studies has performed histological observation of the contents of the FN. Therefore, we aimed to further elucidate an incidentally found FN by gross anatomical, radiological, and histological methods.

## Case presentation

**Figure 1:**
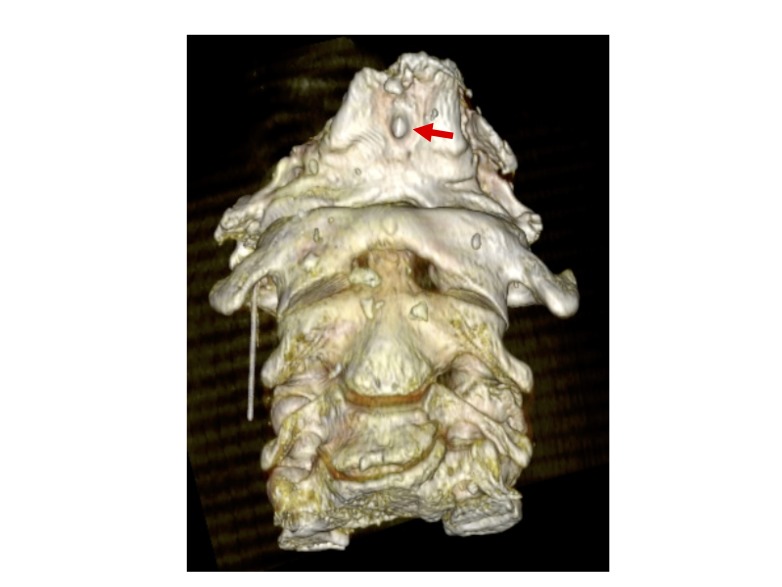
Anterior view of the fossa navicularis (arrow) as seen via 3D-CT. 3D-CT: three-dimensional computed tomography

The FN was found in the occipital bone of a Caucasian male cadaver (74 years old at death) that was examined with CT before routine gross anatomy dissection (Figure 1).

**Figure 2:**
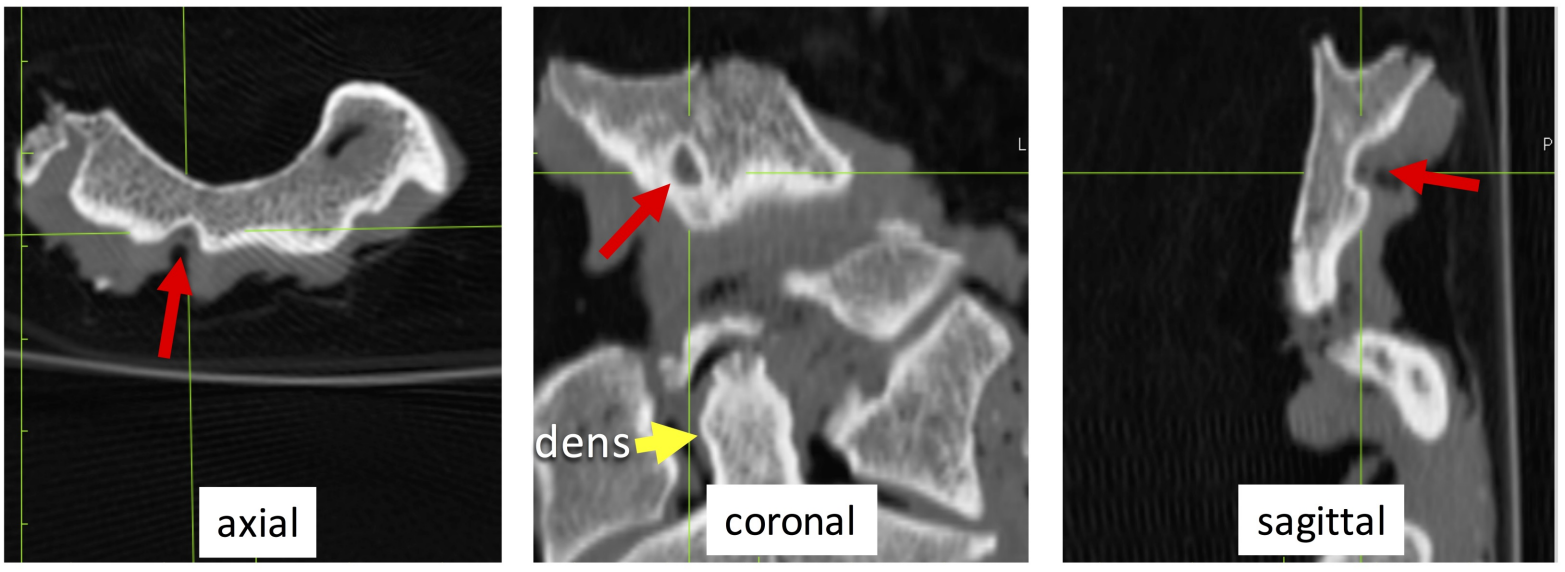
Cross section computed tomography (CT) of the fossa navicularis (red arrow)

CT revealed a shallow concavity on the ventral surface of the occipital part of the clivus, which was located in the midline and anterosuperior to the pharyngeal tubercle. The FN was 4.5 mm in length, 2.5 mm in width, and 2.0 mm in depth (Figure 2).

**Figure 3:**
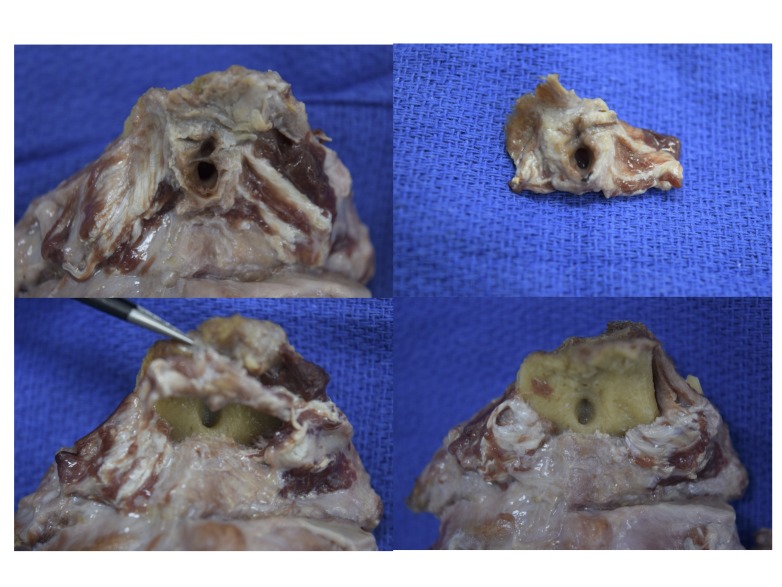
Gross observation of the fossa navicularis

There was no continuity between the FN and the posterior cranial fossa. Next, the overlying soft tissues and tissues invaginating and lining the FN were observed and dissected using a surgical microscope (OPMI CS NC31) (Carl Zeiss, Oberkochen, Germany). No specific structure(s) (e.g., grossly identifiable blood vessels, glands, adipose tissue, cysts, etc.) was found inside or around the fossa (Figure 3).

**Figure 4:**
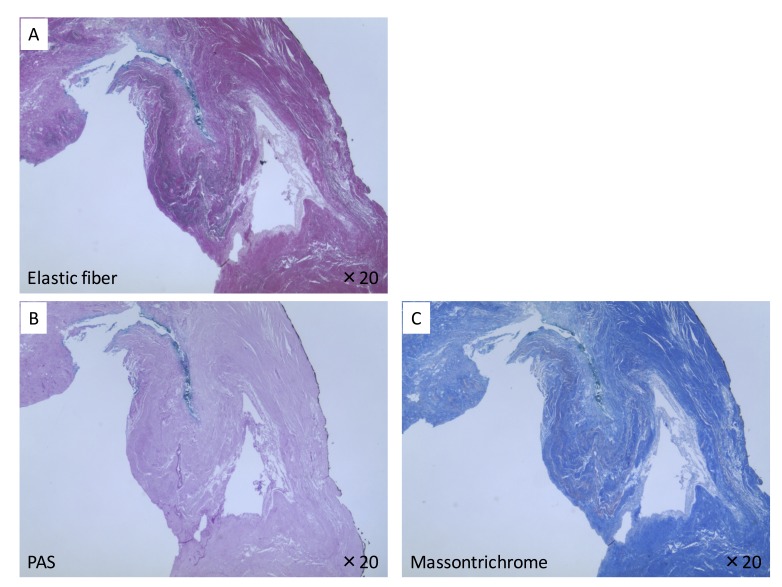
Histological analysis of the soft tissue filling the fossa navicularis. A) Elastic fiber stain; B) periodic acid-Schiff stain; C) Masson's trichrome stain

The soft tissues filling the FN were removed and submitted for histological examination. Hematoxylin and eosin (H&E) stain showed non-specific collagenous and elastic fibers making up the tissue lining. Masson-trichrome stain, elastic fiber stain, and periodic acid-Schiff stain (PAS) showed the superficial part of the overlying tissue, which lay on the fossa, was composed of loose connective tissue with a mixture of collagen and elastic fibers and a vascular matrix including arteries, veins, and capillaries. Less vasculature tissue and elastic fibers filled with collagen fibers were found in the surrounding tissues of the fossa (Figure 4).

There was no evidence of the lymphoid or glandular tissue in any of the histologically analyzed tissues. 

## Discussion

The FN has been termed by various authors as the fossa navicularis magna, medial basal fossa, fossa pharyngea, large pharyngeal fossa, canalis basilaris medianus, keyhole defect, and longitudinal or transverse segmentation defects [1]. Of them all, our literature review found that the FN has been used most frequently with some making distinctions between the FN and the median basal canal [2, 4-5]. According to Currarino [5], the FN is a rare anatomic variant of the basiocciput and is a subtype of the canalis basilaris medianus (CBM) [6]. This author referred to the FN as an incomplete form of the CBM.

The prevalence of the FN is reported from 0.9 to 5.3% and measurements vary from 1.1 mm to 5.5 mm in depth, 1.79 mm to 13 mm in length, and 1.5 mm to 8 mm in width [2, 7-9]. According to Cankal, et al., 2.9% (14/492) of dry human skulls had an FN of greater than or equal to 2 mm in depth and an FN was identified in 3.0 % (16/525) of patients on CT [2].

Generally, two theories have been suggested concerning the embryology of the FN. One theory suggests that all canals and foramina seen on the surface of the basiocciput are vascular in origin and are similar to the basivertebral foramina of the vertebral bodies [8]. However, another theory suggests that the FN represents remnants of the cephalic end of the notochordal canal [9]. According to Staderini [9], the CBM bifurcates have a double origin, the superior canal is vascular in origin and the rest of the defect is related to the notochordal canal. Histological findings of the present case demonstrated that the tissue lining the FN was composed of loose connective tissue with a mixture of collagen and elastic fibers and a vascular matrix. The existence of the vascular matrix might support the former theory of the embryology but does not support a notochordal etiology for the development of the FN. In addition, several MRI studies have described the FN as being filled with lymphoid tissue of the nasopharyngeal tonsils but have made these comments without histology [2-4]. Our study showed soft tissue in the fossa but no lymphoid tissue was found within it.

From the clinical perspective, the differential diagnosis of the FN should include local or metastatic tumor, adenoid retention cyst, Rathke’s pouch cyst, adenoid hypertrophy, and Tornwaldt’s cyst [2, 4]. Knowledge of this bony variation might promote a better diagnosis of diseases in this region [2].

## Conclusions

Review of literature did not show any histological observation of the FN and such analysis offers an interesting window into the true nature of tissues related to this bony depression. Further studies with histological analysis might help better clarify the structure of the FN in a larger series of specimens.
